# Safety and efficacy of abemaciclib plus endocrine therapy in older patients with hormone receptor-positive/human epidermal growth factor receptor 2-negative advanced breast cancer: an age-specific subgroup analysis of MONARCH 2 and 3 trials

**DOI:** 10.1007/s10549-020-06029-y

**Published:** 2021-01-03

**Authors:** Matthew P. Goetz, Meena Okera, Hans Wildiers, Mario Campone, Eva-Maria Grischke, Luis Manso, Valérie A. M. André, Nadia Chouaki, Belén San Antonio, Masakazu Toi, George W. Sledge

**Affiliations:** 1 Department of Oncology, Mayo Clinic, 200 First St. S.W, Rochester, MN 55905 USA; 2 Adelaide Cancer Center, Adelaide, Australia; 3 Department of General Medical Oncology, University Hospitals Leuven, Leuven, Belgium; 4 Institut de Cancerologie de L’Ouest-René Gauducheau, Saint Herblain, France; 5 Women’s Hospital, University Hospital Tübingen, Tübingen, Germany; 6 12 de Octubre University Hospital, Madrid, Spain; 7 Eli Lilly and Company, Paris, France; 8 Eli Lilly and Company, Madrid, Spain; 9 Breast Cancer Unit, Kyoto University Hospital, Kyoto University, Kyoto, Japan; 10 Stanford University School of Medicine, Stanford, CA USA

**Keywords:** Abemaciclib, Age, HR+, HER2−, Metastatic breast cancer, Endocrine therapy

## Abstract

**Purpose:**

Abemaciclib in combination with endocrine therapy (ET) has demonstrated significant efficacy benefits in HR+ , HER2− advanced breast cancer patients in the Phase 3 studies MONARCH 2 (fulvestrant as ET) and MONARCH 3 (letrozole or anastrozole as ET). Here, we report age-specific safety and efficacy outcomes.

**Methods:**

Exploratory analyses of MONARCH 2 and 3 were performed for 3 age groups (<65, 65–74, and ≥75 years). For safety, data were pooled from both studies; for efficacy, a subgroup analysis of PFS was performed for each trial independently.

**Results:**

Pooled safety data were available for 1152 patients. Clinically relevant diarrhea (Grade 2/3) was higher in older patients receiving abemaciclib + ET (<65, 39.5%; 65–74, 45.2%; ≥75, 55.4%) versus placebo + ET (<65, 6.8%; 65–74, 4.5%; ≥75, 16.0%). Nausea, decreased appetite, and venous thromboembolic events were all moderately higher in older patients. Neutropenia (Grade ≥ 3) did not differ as a function of age in the abemaciclib + ET arm (<65, 25.8%; 65–74, 27.4%; ≥75, 18.1%). Dose adjustments and discontinuation rates were slightly higher in older patients. Abemaciclib + ET improved PFS compared with placebo + ET independent of patient age, with no significant difference in abemaciclib treatment effect between the 3 age groups (MONARCH 2: interaction *p*-value, 0.695; MONARCH 3: interaction *p*-value, 0.634). Estimated hazard ratios ranged from 0.523–0.633 (MONARCH 2) and 0.480–0.635 (MONARCH 3).

**Conclusions:**

While higher rates of adverse events were reported in older patients, they were manageable with dose adjustments and concomitant medication. Importantly, a consistent efficacy benefit was observed across all age groups.

**Clinical trial registration:**

ClinicalTrials.gov: NCT02107703 (first posted April 8, 2014) and NCT02246621 (first posted September 23, 2014).

## Introduction

Breast cancer is the leading cancer diagnosis in women, and a significant proportion of these patients are 65 years of age or older [1, 2]. In the United States from 2013 to 2017, the ≥65 age group accounted for almost 45% of all new breast cancer diagnoses (age 65–74, 25.5%; 75–84, 13.6%; >84, 5.4%) and two-thirds of breast cancer deaths (age 65–74, 23.4%; 75–84, 19.7%; >84, 17.2%) [3]. Specifically, hormone receptor-positive (HR+), human epidermal growth factor receptor 2-negative (HER2−) breast cancer subtype has the highest incidence rate per 100,000 across age groups [4], and the majority of HR+, HER2− subtypes occur in older women [5]. Older patients tend to have a higher incidence of comorbidities, including vascular, gastrointestinal, metabolic, nutritional, and renal disorders, and increased risk for treatment-related toxicities like gastrointestinal and renal toxicities, which has relevance for quality of life [2, 6, 7]. The underrepresentation of older patients in clinical trials may reflect lower enrollment due to perceived risk increase for treatment-related adverse events (TEAEs), and/or limitations placed on eligibility due to comorbidities or prior concomitant medications and potential for drug–drug interactions [8]. These points underscore the need for better information on age-specific efficacy, safety, and drug tolerability, particularly in patients older than 75 years.

Current treatment guidelines support the use of endocrine therapy (ET) in combination with a cyclin-dependent kinase (CDK) 4 and 6 inhibitor for treatment of HR+, HER2− advanced or metastatic breast cancer [9–12]. Three CDK4 and 6 inhibitors are now FDA and EMA approved in combination with ET as initial endocrine-based therapy or after progression on ET for this indication [13, 14]. Of these, abemaciclib is approved in combination with letrozole or anastrozole [15] as initial treatment, and in combination with fulvestrant after progression on ET [16]. In MONARCH 3, abemaciclib + ET demonstrated statistically significant and clinically meaningful progression-free survival (PFS) improvement compared to ET alone [median 28.18 vs. 14.76 months; hazard ratio (HR), 0.540; *P* = 0.000002] [17]. For MONARCH 2, both PFS (median 16.4 vs. 9.3 months; HR, 0.553; *P* < 0.001) and OS (median 46.7 vs. 37.3 months; HR, 0.757; *P* = 0.01) were longer in the abemaciclib arm compared to placebo [16, 18]. Abemaciclib was also the first CDK4 and 6 inhibitor with approval as monotherapy in patients with refractory HR+, HER2− metastatic breast cancer (MONARCH 1) [19].

In a recent safety analysis of MONARCH 2 and 3 [20], the most frequent TEAE in patients taking abemaciclib was diarrhea, with clinically significant diarrhea (Grade ≥ 2) reported in approximately 43% of patients, and a median onset of 1 week. Grade 3/4 neutropenia occurred in approximately 25% of patients receiving abemaciclib across studies. However, abemaciclib discontinuation due to these AEs occurred in <3% of patients, indicating these AEs could be successfully managed by dose adjustment and/or use of supportive medication. The safety profile together with the established efficacy of abemaciclib supports a favorable clinical benefit/risk ratio overall in patients with HR+, HER2− advanced breast cancer. Despite this, guidance on the use of abemaciclib in older patient populations is lacking. To address this knowledge gap, here, we report an age-specific analysis of the safety and efficacy of abemaciclib in older patient populations from the Phase 3 studies MONARCH 2 and MONARCH 3.

## Methods

### Study design

Data from the Phase 3 trials MONARCH 2 (NCT02107703) and MONARCH 3 (NCT02246621), which studied abemaciclib in combination with ET in patients with HR+, HER2− ABC were included in this analysis. MONARCH 2 was a global, double-blind, Phase 3 study that included women with HR+, HER2− locally advanced or metastatic breast cancer who experienced disease progression while receiving neoadjuvant or adjuvant ET, ≤12 months after adjuvant ET, or while receiving first-line ET for ABC [16]. MONARCH 3 included post-menopausal women with HR+, HER2− locoregionally recurrent or metastatic breast cancer who had no prior systemic therapy in the advanced setting [15]. Both studies were reviewed and approved by ethical and institutional review boards. All patients provided written informed consent prior to study enrollment. The trials were conducted in accordance with the 1964 Declaration of Helsinki and its later amendments or comparable ethical standards. Full study design details and eligibility criteria have been published [15, 16].

### Treatments

Patients in MONARCH 2 received abemaciclib or placebo, orally, twice daily (BID) and fulvestrant 500 mg by intramuscular injection on days 1 and 15 of Cycle 1 and on day 1 of subsequent 28-day cycles. Patients enrolled at study initiation received abemaciclib at 200 mg; however, the protocol was amended after review of safety data and dose reduction rates to reduce starting dose to 150 mg for new patients. Patients who were receiving 200 mg underwent a mandatory dose reduction to 150 mg [16]. Patients in MONARCH 3 received abemaciclib 150 mg orally BID (or placebo) plus either anastrozole 1 mg or letrozole 2.5 mg, both orally once a day. If either abemaciclib or placebo was discontinued, patients were permitted to continue receiving the ET; if the ET required discontinuation, patients were permitted to continue receiving abemaciclib or placebo.

### Outcomes and statistical assessments

Safety and efficacy of abemaciclib were assessed in 3 age subgroups: <65, 65–74, and ≥75 years. While patients >65 are considered older, the ≥75 group is the most representative of a real-world geriatric population with respect to age. Efficacy was evaluated on the intent-to-treat (ITT) population, and safety was assessed in all patients who received ≥1 dose of the study drug. Adverse events (AEs) were graded according to the National Cancer Institute Common Terminology Criteria for Adverse Events (version 4.0). To assess the impact of age on safety and tolerability, data were pooled across studies and summarized using descriptive statistics. Investigator-assessed PFS was analyzed using the Kaplan–Meier method, and the age subgroup analysis of PFS was performed for each trial independently using a Cox-proportional hazard model. Interaction test of age groups by treatment arm was performed at the two-sided 0.05 level.

## Results

### Patients

**Fig. 1:**
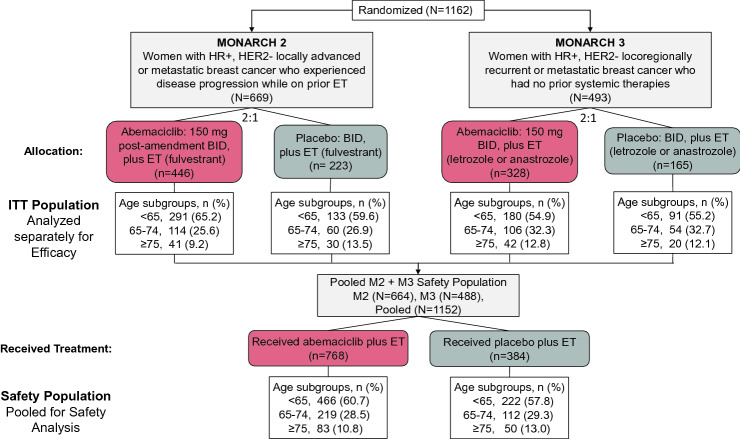
Study design. Data cutoff for MONARCH 2 was Feb 14, 2017; MONARCH 3 data cutoff was Nov 3, 2017. *BID* twice daily, *ET* endocrine therapy, *HR+* hormone receptor-positive, *HER2−* human epidermal growth factor receptor 2 negative, *ITT* intent to treat, *M2* MONARCH 2, *M3* MONARCH 3, *n* number of patients, *N* total population

In MONARCH 2 and MONARCH 3, a total of 1162 patients were randomized to receive treatment. For MONARCH 2, patients were allocated to abemaciclib plus fulvestrant (*n* = 446) or placebo plus fulvestrant (*n* = 223) [16]. At study initiation, patients in the abemaciclib arm received a 200 mg BID dose (*n* = 121, 27.4%) for a median duration of 34 days before a dose reduction to 150 mg or discontinuation. For MONARCH 3, patients were allocated to abemaciclib 150 mg BID plus nonsteroidal AI (either anastrozole or letrozole) (*n* = 328) or placebo plus AI (*n* = 165) (Fig. 1).

Treatment arms for each study were divided into 3 age subgroups: <65, 65–74, and ≥75 years of age. The ITT population age breakdown of MONARCH 2 was 291 (65.2%) patients <65 years, 114 (25.6%) 65–74 years, and 41 (9.2%) ≥75 years of age in the abemaciclib plus fulvestrant arm, and 133 (59.6%) patients <65 years, 60 (26.9%) 65–74 years, and 30 (13.5%) ≥75 years of age in the placebo arm. The ITT population of MONARCH 3 comprised 180 (54.9%) patients <65 years, 106 (32.3%) 65–74 years, and 42 (12.8%) ≥75 years of age in the abemaciclib plus nonsteroidal AI arm, and 91 (55.2%) patients <65 years, 54 (32.7%) 65–74 years, and 20 (12.1%) ≥75 years of age in the placebo arm (Fig. 1).

Distribution of patient baseline disease characteristics was generally well balanced for shared characteristics between the 2 studies, across treatment arms, and between age groups (Table 1, MONARCH 2 and Table 2, MONARCH 3). In both MONARCH 2 and 3, the proportion of patients with an ECOG PS of 1 was generally higher in the 65–74 and ≥75 age groups compared to the <65 age group. Older patients had a higher incidence at baseline of comorbidities related to vascular disorders (mainly hypertension), gastrointestinal disorders (constipation, gastroesophageal reflux disease), cardiac disorders, and metabolism and nutrition disorders (including hypercholesterolemia and hyperglycemia).

**Table 1:** Baseline disease characteristics and comorbidities in MONARCH 2 by age subgroup

Baseline characteristics, *n* (%)	Abemaciclib + ET^a^			Placebo + ET^a^		
	<65	65–74	≥75	<65	65–74	≥75
	*n* = 291	*n* = 114	*n* = 41	*n* = 133	*n* = 60	*n* = 30
ECOG PS^b^						
0	182 (62.5)	64 (56.1)	18 (43.9)	88 (66.2)	30 (50.0)	18 (60.0)
1	104 (35.7)	49 (43.0)	23 (56.1)	45 (33.8)	30 (50.0)	12 (40.0)
Nature of the disease						
Visceral	162 (55.7)	56 (49.1)	27 (65.9)	73 (54.9)	36 (60.0)	19 (63.3)
Bone only	83 (28.5)	32 (28.1)	8 (19.5)	39 (29.3)	13 (21.7)	5 (16.7)
Other	43 (14.8)	26 (22.8)	6 (14.6)	21 (15.8)	11 (18.3)	6 (20.0)
PgR status^c^						
Positive	229 (78.7)	82 (71.9)	28 (68.3)	114 (85.7)	38 (63.3)	19 (63.3)
Negative	55 (18.9)	29 (25.4)	12 (29.3)	18 (13.5)	17 (28.3)	9 (30.0)
ET sensitivity						
No prior ET	5 (1.7)	1 (0.9)	0 (0.0)	1 (0.8)	1 (1.7)	0 (0.0)
Primary resistance	84 (28.9)	20 (17.5)	7 (17.1)	38 (28.6)	13 (21.7)	7 (23.3)
Secondary resistance	199 (68.4)	93 (81.6)	34 (82.9)	94 (70.7)	46 (76.7)	23 (76.7)

Comorbidities, *n* (%)	*n* = 287	*n* = 113	*n* = 41	*n* = 133	*n* = 60	*n* = 30
Vascular disorders^d^	105 (36.6)	64 (56.6)	29 (70.7)	51 (38.3)	38 (63.3)	25 (83.3)
Hypertension	72 (25.1)	60 (53.1)	27 (65.9)	38 (28.6)	35 (58.3)	25 (83.3)
Gastrointestinal disorders	73 (25.4)	33 (29.2)	17 (41.5)	31 (23.3)	23 (38.3)	12 (40.0)
Constipation	21 (7.3)	10 (8.8)	10 (24.4)	7 (5.3)	4 (6.7)	2 (6.7)
Gastroesophageal reflux disease	13 (4.5)	13 (11.5)	5 (12.2)	7 (5.3)	4 (6.7)	5 (16.7)
Nausea	16 (5.6)	2 (1.8)	2 (4.9)	6 (4.5)	3 (5.0)	1 (3.3)
Abdominal pain	8 (2.8)	5 (4.4)	0 (0.0)	3 (2.3)	1 (1.7)	1 (3.3)
Diarrhea	6 (2.1)	8 (7.1)	0 (0.0)	3 (2.3)	4 (6.7)	0 (0.0)
Metabolism and nutrition disorders	57 (19.9)	44 (38.9)	18 (43.9)	29 (21.8)	23 (38.3)	14 (46.7)
Hypercholesterolemia	24 (8.4)	25 (22.1)	13 (31.7)	11 (8.3)	12 (20.0)	6 (20.0)
Hyperglycemia	14 (4.9)	16 (14.2)	3 (7.3)	10 (7.5)	8 (13.3)	4 (13.3)
Decreased appetite	10 (3.5)	3 (2.7)	0 (0.0)	3 (2.3)	4 (6.7)	1 (3.3)
Cardiac disorders	26 (9.1)	11 (9.7)	9 (22.0)	14 (10.5)	14 (23.3)	3 (10.0)
Renal and urinary disorders	13 (4.5)	7 (6.2)	3 (7.3)	11 (8.3)	7 (11.7)	4 (13.3)
Blood creatinine increase	3 (1.0)	4 (3.5)	0 (0.0)	0 (0.0)	0 (0.0)	1 (3.3)

Data given as *n* (%) unless otherwise indicated

*ECOG PS* Eastern Cooperative Oncology Group performance status, *ET* endocrine therapy, *n* number of patients, *PgR* progesterone receptor

^a^Patients in MONARCH 2 received abemaciclib or placebo plus fulvestrant

^b^One patient in the age 65–74 abemaciclib arm had an ECOG PS of 2+

^c^Patients with unknown PgR status not shown

^d^Embolism reported in ≤2.1% of patients in each age group

**Table 2:** Baseline disease characteristics and comorbidities in MONARCH 3 by age subgroup

Baseline characteristics, *n* (%)	Abemaciclib + ET^a^			Placebo + ET^a^		
	<65	65–74	≥75	<65	65–74	≥75
	*n* = 180	*n* = 106	*n* = 42	*n* = 91	*n* = 54	*n* = 20
ECOG PS						
0	115 (63.9)	62 (58.5)	15 (35.7)	60 (65.9)	34 (63.0)	10 (50.0)
1	65 (36.1)	44 (41.5)	27 (64.3)	31 (34.1)	20 (37.0)	10 (50.0)
Disease setting						
De novo metastatic	73 (40.6)	42 (39.6)	20 (47.6)	28 (30.8)	25 (46.3)	8 (40.0)
Metastatic recurrent	104 (57.8)	58 (54.7)	20 (47.6)	59 (64.8)	29 (53.7)	11 (55.0)
Locoregionally recurrent	3 (1.7)	6 (5.7)	2 (4.8)	4 (4.4)	0 (0.0)	1 (5.0)
Nature of the disease						
Visceral	95 (52.8)	54 (50.9)	23 (54.8)	47 (51.6)	31 (57.4)	11 (55.0)
Bone only	37 (20.6)	27 (25.5)	6 (14.3)	25 (27.5)	14 (25.9)	0 (0.0)
Other	48 (26.7)	25 (23.6)	13 (31.0)	19 (20.9)	9 (16.7)	9 (45.0)
PgR status^b^						
Positive	139 (77.2)	80 (75.5)	36 (85.7)	71 (78.0)	40 (74.1)	16 (80.0)
Negative	41 (22.8)	23 (21.7)	6 (14.3)	20 (22.0)	12 (22.2)	4 (20.0)

Comorbidities, *n* (%)	*n* = 179	*n* = 106	*n* = 42	*n* = 89	*n* = 52	*n* = 20
Vascular disorders^c^ Hypertension	52 (29.1) 37 (20.7)	61 (57.5) 57 (53.8)	27 (64.3) 25 (59.5)	22 (24.7) 19 (21.3)	27 (51.9) 21 (40.4)	12 (60.0) 12 (60.0)
Gastrointestinal disorders Constipation Gastroesophageal reflux disease Nausea Abdominal pain Diarrhea	33 (18.4) 7 (3.9) 4 (2.2) 7 (3.9) 4 (2.2) 1 (0.6)	31 (29.2) 10 (9.4) 7 (6.6) 4 (3.8) 0 (0.0) 3 (2.8)	15 (35.7) 3 (7.1) 6 (14.3) 4 (9.5) 0 (0.0) 0 (0.0)	20 (22.5) 5 (5.6) 7 (7.9) 3 (3.4) 1 (1.1) 0 (0.0)	17 (32.7) 5 (9.6) 4 (7.7) 1 (1.9) 0 (0.0) 1 (1.9)	6 (30.0) 1 (5.0) 2 (10.0) 0 (0.0) 0 (0.0) 0 (0.0)
Metabolism and nutrition disorders Hypercholesterolemia Hyperglycemia Decreased appetite	40 (22.3) 16 (8.9) 13 (7.3) 3 (1.7)	55 (51.9) 26 (24.5) 21 (19.8) 3 (2.8)	17 (40.5) 9 (21.4) 10 (23.8) 2 (4.8)	21 (23.6) 5 (5.6) 7 (7.9) 3 (3.4)	21 (40.4) 10 (19.2) 7 (13.5) 2 (3.8)	11 (55.0) 5 (25.0) 4 (20.0) 1 (5.0)
Cardiac disorders	6 (3.4)	11 (10.4)	11 (26.2)	2 (2.2)	8 (15.4)	4 (20.0)
Renal and urinary disorders	7 (3.9)	8 (7.5)	5 (11.9)	5 (5.6)	6 (11.5)	2 (10.0)
Blood creatinine increase	0 (0.0)	2 (1.9)	2 (4.8)	0 (0.0)	0 (0.0)	0 (0.0)

Data given as *n* (%) unless otherwise indicated

*ECOG PS* Eastern Cooperative Oncology Group performance status, *ET* endocrine therapy, *n* number of patients, *PgR* progesterone receptor

^a^Patients in MONARCH 3 received abemaciclib or placebo plus either letrozole or anastrozole

^b^Patients with unknown PgR status not shown

^c^Embolism reported in ≤3.2% of patients in each age group

### Safety

Pooled safety data were available for 1152 patients treated in the MONARCH 2 and 3 trials (MONARCH 2, *N* = 664; MONARCH 3, *N* = 488) (Fig. 1). Pooled across treatment arms, this included 688 (59.7%) <65; 331 (28.7%) 65–74; and 133 (11.5%) ≥75 years.

The most common TEAEs in the abemaciclib + ET arm (any age group) from the pooled analysis of MONARCH 2 and MONARCH 3 are shown in Table 3. The most frequent any grade TEAE was diarrhea, followed by neutropenia, nausea, and fatigue. The incidence of diarrhea was similar in abemaciclib-treated patients across all age groups (~85%). In the abemaciclib arm, other gastrointestinal toxicities such as nausea and decreased appetite were moderately higher (by 10–20%) in the 2 older subgroups. In contrast, abdominal pain, vomiting, and constipation were not increased in the older subgroups compared to <65 group. Fatigue was slightly higher in the 2 abemaciclib-treated older age groups (<65, 34.8%; 65–74, 48.4%, ≥75, 51.8%). Hematological toxicities including neutropenia, anemia, and leukopenia were higher in the abemaciclib + ET arm compared to the placebo arm, but no differences in incidence were seen across age groups. Increased blood creatinine was higher in the abemaciclib arm and was also higher in both the 65–74 and ≥75 age groups compared to the <65 group.

**Table 3:** Pooled TEAEs from MONARCH 2 and MONARCH 3 occurring in ≥20% patients in any age group in the abemaciclib + ET arm

TEAE, any grade, n (%)^b^	Abemaciclib + ET^a^			Placebo + ET^a^		
	<65	65–74	≥75	<65	65–74	≥75
	(*n* = 466)	(*n* = 219)	(*n* = 83)	(*n* = 222)	(*n* = 112)	(*n* = 50)
Diarrhea	396 (85.0)	183 (83.6)	71 (85.5)	57 (25.7)	33 (29.5)	17 (34.0)
Neutropenia	215 (46.1)	106 (48.4)	25 (30.1)	8 (3.6)	4 (3.6)	0 (0.0)
Nausea	181 (38.8)	114 (52.1)	39 (47.0)	50 (22.5)	24 (21.4)	10 (20.0)
Fatigue	162 (34.8)	106 (48.4)	43 (51.8)	66 (29.7)	34 (30.4)	14 (28.0)
Abdominal pain	169 (36.3)	67 (30.6)	22 (26.5)	38 (17.1)	11 (9.8)	7 (14.0)
Anemia	127 (27.3)	79 (36.1)	25 (30.1)	8 (3.6)	9 (8.0)	4 (8.0)
Vomiting	124 (26.6)	63 (28.8)	26 (31.3)	26 (11.7)	16 (14.3)	2 (4.0)
Decreased appetite	95 (20.4)	75 (34.2)	33 (39.8)	26 (11.7)	13 (11.6)	5 (10.0)
Leukopenia	119 (25.5)	58 (26.5)	20 (24.1)	7 (3.2)	1 (0.9)	0 (0.0)
Alopecia	102 (21.9)	43 (19.6)	14 (16.9)	14 (6.3)	7 (6.3)	1 (2.0)
Headache	109 (23.4)	37 (16.9)	8 (9.6)	50 (22.5)	9 (8.0)	1 (2.0)
Blood creatinine increase	42 (9.0)	59 (26.9)	18 (21.7)	2 (0.9)	4 (3.6)	2 (4.0)
Constipation	59 (12.7)	45 (20.5)	13 (15.7)	29 (13.1)	17 (15.2)	7 (14.0)
Cough	49 (10.5)	47 (21.5)	11 (13.3)	28 (12.6)	14 (12.5)	3 (6.0)
Dyspnea	36 (7.7)	35 (16.0)	17 (20.5)	20 (9.0)	11 (9.8)	5 (10.0)

*TEAE* treatment-emergent adverse event, *ET* endocrine therapy, *n* number of patients

^a^Patients in MONARCH 2 received abemaciclib plus fulvestrant; patients in MONARCH 3 received abemaciclib plus either letrozole or anastrozole

^b^ Ordered by decreasing frequency (any grade) of combined age groups (total) in the abemaciclib + ET arm

^c^There were no Grade 4 diarrhea events

Select AEs of clinical interest are reported in more detail in Table 4. Clinically relevant diarrhea (Grade 2/3) occurred more frequently in the 2 older groups (<65, 39.5%; 65–74, 45.2%; ≥75, 55.4%). Of note, in the placebo arm, Grade 2/3 diarrhea was more common in the ≥75 group compared to the <65 group (<65, 6.8%; 65–74, 4.5%; ≥75, 16.0%). Neutropenia was the most common Grade ≥ 3 AE (Table 4); however, incidence of Grade ≥ 3 neutropenia did not differ as a function of age in either the abemaciclib arm (<65, 25.8%; 65–74, 27.4%; ≥75, 18.1%) or placebo arm. Hepatic events (increased ALT, AST, ALP, and bilirubin levels) in the abemaciclib arm were reported with similar, or lower, incidence in the 65–74 and ≥75 subgroups compared to the <65 age group. Interstitial lung disease (ILD)/pneumonitis was infrequent and reported at similar frequency (~3.5%) across age groups in the abemaciclib arm, with <1.5% of cases Grade ≥ 3 (Table 4). The incidence of venous thromboembolic events (VTEs), including pulmonary embolism or deep vein thrombosis (DVT), was consistent in the 2 younger age groups (<65; 4.1%, 65–74; 5.0%); in contrast, the incidence of VTEs was more common in patients aged ≥75 (≥75; 11 of 83 patients, 13.3%).

**Table 4:** Selected AEs pooled from MONARCH 2 and MONARCH 3

AE, *n* (%)	Abemaciclib + ET^a^			Placebo + ET^a^		
	<65	65–74	≥75	<65	65–74	≥75
	(*n* = 466)	(*n* = 219)	(*n* = 83)	(*n* = 222)	(*n* = 112)	(*n* = 50)
Diarrhea						
Any grade	396 (85.0)	183 (83.6)	71 (85.5)	57 (25.7)	33 (29.5)	17 (34.0)
Grade 2/3	184 (39.5)	99 (45.2)	46 (55.4)	15 (6.8)	5 (4.5)	8 (16.0)
Grade 3^b^	46 (9.9)	28 (12.8)	16 (19.3)	1 (0.5)	0 (0.0)	2 (4.0)
Neutropenia						
Any grade	215 (46.1)	106 (48.4)	25 (30.1)	8 (3.6)	4 (3.6)	0 (0.0)
Grade ≥ 3	120 (25.8)	60 (27.4)	15 (18.1)	4 (1.8)	2 (1.8)	0 (0.0)
ALT increase						
Any grade	76 (16.3)	33 (15.1)	7 (8.4)	15 (6.8)	6 (5.4)	3 (6.0)
Grade ≥ 3	23 (4.9)	12 (5.5)	4 (4.8)	5 (2.3)	1 (0.9)	1 (2.0)
AST increase						
Any grade	68 (14.6)	34 (15.5)	7 (8.4)	17 (7.7)	7 (6.3)	3 (6.0)
Grade ≥ 3	13 (2.8)	7 (3.2)	2 (2.4)	4 (1.8)	3 (2.7)	1 (2.0)
Blood ALP increase						
Any grade	20 (4.3)	15 (6.8)	4 (4.8)	8 (3.6)	3 (2.7)	2 (4.0)
Grade ≥ 3	3 0.6)	3 (1.4)	1 (1.2)	0 (0.0)	0 (0.0)	1 (2.0)
Blood bilirubin increase						
Any grade	7 (1.5)	5 (2.3)	1 (1.2)	2 (0.9)	1 (0.9)	0 (0.0)
Grade ≥ 3	5 (1.1)	2 (0.9)	0 (0.0)	0 (0.0)	0 (0.0)	0 (0.0)
VTE events^c^						
Any grade Grade ≥ 3	19 (4.1) 9 (1.9)	11 (5.0) 6 (2.7)	11 (13.3) 4 (4.8)	1 (0.5) 1 (0.5)	1 (0.9) 1 (0.9)	1 (2.0) 0 (0.0)
ILD/pneumonitis events^d, e^						
Any grade	16 (3.4)	7 (3.2)	3 (3.6)	2 (0.9)	0 (0.0)	0 (0.0)
Grade ≥ 3	4 (0.9)	3 (1.4)	0 (0.0)	0 (0.0)	0 (0.0)	0 (0.0)

*AE* adverse event, *ALP* alkaline phosphatase, *ALT* alanine aminotransferase, *AST* aspartate aminotransferase, *ET* endocrine therapy, *ILD* interstitial lung disease, *n* number of patients, *VTE* venous thromboembolic event

^a^Patients in MONARCH 2 received abemaciclib plus fulvestrant; patients in MONARCH 3 received abemaciclib plus either letrozole or anastrozole

^b^There were no Grade 4 diarrhea events

^c^There were 3 fatal cases of VTEs in MONARCH 2 and 3, 2 in the <65 age group and 1 in the 65–74 age group

^d^As used here, “ILD/Pneumonitis Events” is a consolidated term incorporating the MedDRA preferred terms “pneumonitis,” “interstitial lung disease,” “organising pneumonia,” and “pulmonary fibrosis”

^e^There were 2 fatal cases in MONARCH 2, 1 in the <65 age group and 1 in the 65–74 age group. There was 1 fatal case in MONARCH 3, in the 65–74 age group

In the pooled analysis of MONARCH 2 and MONARCH 3, patients treated with abemaciclib in the 65–74 and ≥75 subgroups had more dose adjustments compared to patients <65 years of age (Table 5). Abemaciclib dose omissions and reductions were mainly due to AEs. The most frequent AEs leading to dose adjustments were diarrhea and neutropenia. The incidence of dose omissions due to the AE of diarrhea was slightly higher in the 2 older age groups (<65, 12.7%; 65–74, 22.4%; ≥75, 30.1%). However, there was only a modest increase in dose reductions due to the AE of diarrhea in those groups as compared to the <65 patients (<65, 14.6%; 65–74, 18.7%; ≥75, 22.9%).

**Table 5:** AE management: pooled analysis of MONARCH 2 and MONARCH 3 dose adjustments, study treatment discontinuations, and concomitant medications

	Abemaciclib + ET		
	<65 (*n* = 466)	65–74 (*n* = 219)	≥75 (*n* = 83)
**Dose adjustments,** ***n* (%)**			
Pts with ≥ 1 dose adjustment	294 (63.1)	163 (74.4)	63 (75.9)
Pts with ≥ 1 dose omission	259 (55.6)	151 (68.9)	60 (72.3)
Dose omission due to AEs	225 (48.3)	143 (65.3)	58 (69.9)
Diarrhea	59 (12.7)	49 (22.4)	25 (30.1)
Neutropenia	75 (16.1)	46 (21.0)	8 (9.6)
Pts with ≥ 1 dose reduction	199 (42.7)	125 (57.1)	46 (55.4)
Dose reduction due to AEs	175 (37.6)	120 (54.8)	46 (55.4)
Diarrhea	68 (14.6)	41 (18.7)	19 (22.9)
Neutropenia	52 (11.2)	29 (13.2)	5 (6.0)
**Study treatment discontinuations,** ***n*** **(%)**			
Pts discontinued all study treatment due to AE	41 (8.8)	31 (14.2)	20 (24.1)
Diarrhea	2 (0.43)	4 (1.83)	4 (4.82)
Lung infection	5 (1.1)	3 (1.4)	0 (0.0)
ALT increase	4 (0.9)	3 (1.4)	0 (0.0)
**Concomitant medications pts with ≥ 1, *n*** **(%)**			
Antidiarrheals	342 (73.4)	157 (71.7)	60 (72.3)
Antiemetics	54 (11.6)	45 (20.5)	17 (20.5)
G-CSF/GM-CSF	30 (6.4)	14 (6.4)	2 (2.4)

*AE* adverse events, *ALT* alanine amino transferase, *ET* endocrine therapy, *G-CSF* granulocyte colony-stimulating factor, *GM-CSF* granulocyte/macrophage colony-stimulating factor, *n* number of patients, *Pts* patients

Discontinuation of all study treatment due to AEs was higher in the 65–74 and ≥75 subgroups compared to the <65 group (Table 5) (<65, 8.8%; 65–74, 14.2%; ≥75, 24.1%). The most common AE leading to study treatment discontinuation in all age groups combined was diarrhea (*n* = 10, 1.3%), followed by lung infection and ALT increased, which accounted for a small percentage of patients (Table 5). Of these, only diarrhea as the reason for study discontinuation was notably higher in the 2 older subgroups (<65, 0.4%; 65–74, 1.8%; ≥75, 4.8%) (Table 5). Neutropenia was not a cause for study treatment discontinuation for any patient in either the 65–74 or ≥75 age group, and only accounted for 3 patients (0.6%) discontinuing study treatment in the <65 age group.

In MONARCH 2, the age breakdown of patients initially receiving the 200 mg BID dose in the abemaciclib arm was 77 (26.5%) in the <65 age group, 30 (26.3%) 65–74, and 14 (34.1%) ≥75 years. Patients starting with the 200 mg BID dose required more dose adjustments due to AEs in all age groups. The percentage of patients with dose reductions due to AEs starting at 200 mg BID abemaciclib by age group was <65, 51.9%; 65–74, 60.0%; ≥75, 71.4%, compared to patients starting at 150 mg abemaciclib <65, 32.4%; 65–74, 50.6%; ≥75, 40.7%. Dose reductions due to diarrhea occurred more frequently in MONARCH 2 patients starting on 200 mg BID abemaciclib (<65, 27.3%; 65–74, 33.3%; ≥75, 50.0%) compared to those receiving 150 mg (<65, 11.9%; 65–74, 20.5%; ≥75, 11.1%). In addition, discontinuation of all study treatment due to AEs was also skewed with more cases occurring in the MONARCH 2 patients starting at the 200 mg BID dose (<65, 13.0%; 65–74, 16.7%, ≥75, 21.4%) versus in those starting at the 150 mg dose (<65, 4.8%; 65–74, 6.0%; ≥75, 18.5%). Management of AEs was accomplished primarily through dose adjustments and the use of antidiarrheals, antiemetics, and colony-stimulating factors (Table 5). The use of antidiarrheals and colony-stimulating factors was not different between age groups; however, there was an increase in the use of antiemetics in the 65–74 and ≥75 groups compared to patients <65 years (<65, 11.6%; 65–74, 20.5%; ≥75, 20.5%).

### Efficacy

**Fig. 2:**
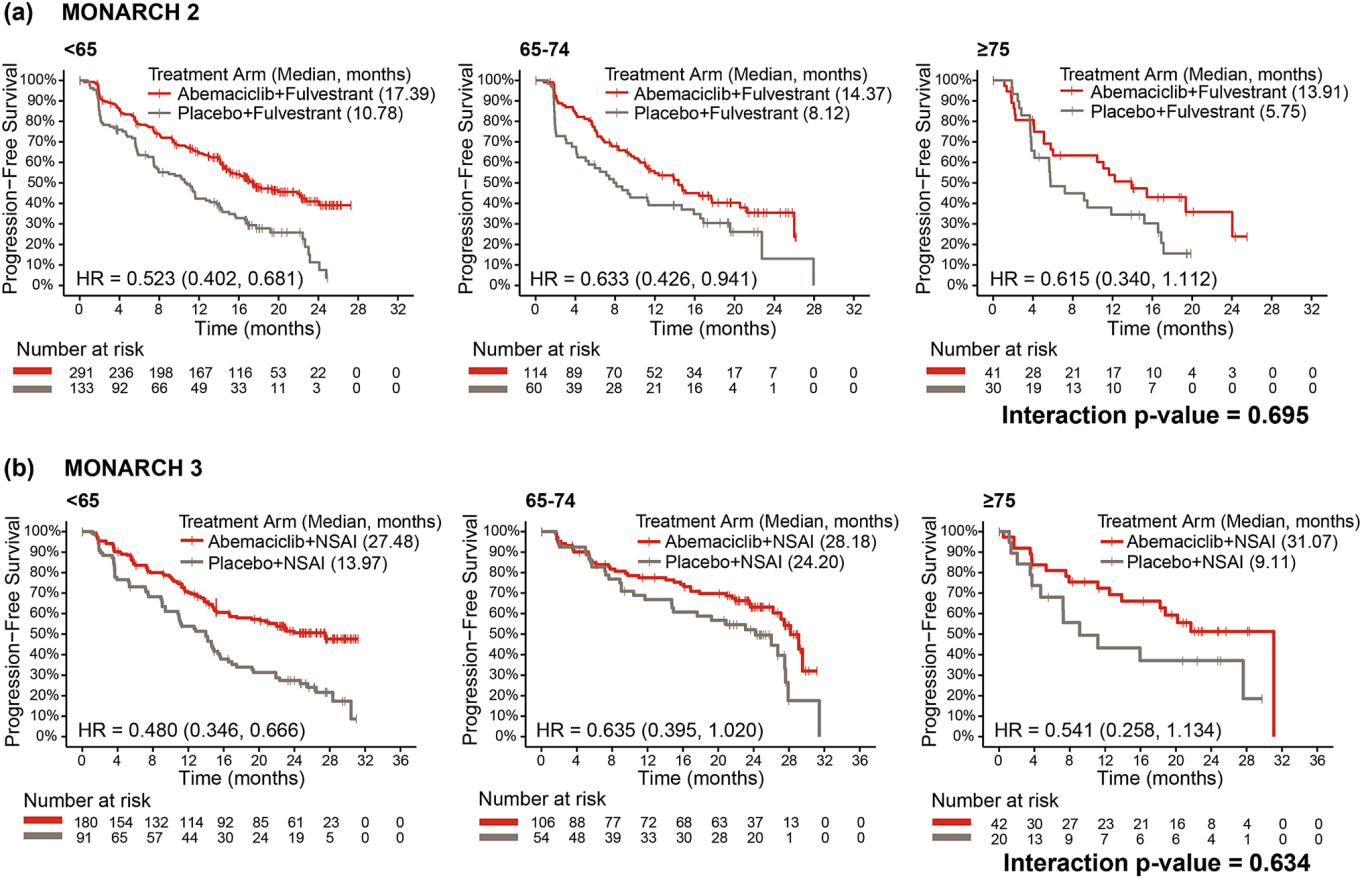
PFS by age subgroup in **a** MONARCH 2 and **b** MONARCH 3. *HR* hazard ratio, *NSAI* nonsteroidal aromatase inhibitor

Consistent with the ITT population in MONARCH 2 and 3, PFS benefit with abemaciclib + ET was observed across age subgroups. (Fig. 2). There was no significant difference in abemaciclib treatment effect between the 3 age subgroups (MONARCH 2: interaction *p*-value, 0.695; MONARCH 3: interaction *p*-value, 0.634). In MONARCH 2 (Fig. 2a), estimated median PFS in the abemaciclib + ET arm compared to the placebo + ET arm was 17.4 months versus 10.8 (HR, 0.523; 95% CI, 0.402–0.681) in the <65 age group, 14.4 months versus 8.1 (HR, 0.633; 95% CI, 0.426–0.941) in 65–74 age group, and 13.9 months versus 5.8 (HR, 0.615; 95% CI, 0.340–1.112) in the ≥75 age group. In MONARCH 3 (Fig. 2b), median PFS in the abemaciclib + ET arm compared to the placebo + ET arm was 27.5 months versus 14.0 (HR, 0.480; 95% CI, 0.346–0.666) in the <65 age group, 28.2 months versus 24.2 (HR, 0.635; 95% CI, 0.395–1.020) in the 65–74 age group, and 31.1 months versus 9.1 (HR, 0.541; 95% CI, 0.258–1.134) in the ≥75 age group.

## Discussion

This post hoc analysis of MONARCH 2 and 3 is the first to report age-specific safety, tolerability, and efficacy outcomes of abemaciclib + ET in women with HR+, HER2− ABC. By pooling the data across these two randomized, placebo-controlled, Phase 3 studies, we were able to increase the number of patients 75 years or older in our analysis, an age group that is more representative of a real-world geriatric population with respect to age. However, the proportion of patients in that demographic was still low, accounting for only 11.5% of the pooled safety population. Subjects enrolled in clinical trials may not be representative of the general population seen in clinical practice [5]. This is particularly true in the case of older patient populations, where narrow eligibility criteria often exclude all but the healthiest older patients [2]. MONARCH 2 and 3 studies were designed to explore safety in a broad age population; thus, the protocols did not include a comprehensive geriatric assessment for older patients. As a result, we are unable to fully characterize the fitness of the older patients included in this pooled analysis. More recently, the use of geriatric assessments in clinical trials enrolling older patients as standard practice has been suggested [21, 22]. The relevance of comprehensive geriatric assessments to guide CDK4 and 6 inhibitor treatment plans in older patients with breast cancer has also been noted [2].

There are also some limitations due to study differences: MONARCH 2 enrolled a more pretreated study population [16], and each study incorporated different ETs. Although outcome analysis and conclusions presented here apply to the abemaciclib dose approved in combination with ET (150 mg BID), the fact that MONARCH 2 initially enrolled patients on 200 mg BID abemaciclib, which is the approved monotherapy dose [19], added some complexity to the safety analysis. Since the median number of days receiving 200 mg of abemaciclib before dose reduction or discontinuation was 34 days, the impact was limited to toxicities that appear early in treatment, like diarrhea. Where possible, we have endeavored to point out instances where this higher dose may confound the interpretation of the pooled safety data.

As expected with abemaciclib, the most frequent TEAEs were gastrointestinal toxicities. Nausea and decreased appetite were moderately increased in the 2 older subgroups, as was use of antiemetics. This is not unexpected considering patients in the 65–74 and ≥75 age groups had a higher incidence of pre-existing gastrointestinal and metabolism and nutrition disorders that may have been aggravated by the exposure to abemaciclib. Although the incidence of any grade diarrhea was similar in patients treated with abemaciclib + ET across age groups, more patients age ≥75 experienced clinically significant (Grade 2/3) diarrhea compared to the other age groups; the use of antidiarrheals was not different between age groups. Incidence of clinically significant diarrhea was highest in the ≥75 age group regardless of treatment arm. Dose reductions due to diarrhea in the ≥75 age group were influenced by the starting dose; 50% of patients starting at 200 mg required dose reductions due to diarrhea compared to only 11% of patients starting at 150 mg. Therefore, while the overall safety findings were consistent across the pre-amendment and post-amendment populations, there were some differences in toxicities expected to occur early in the course of treatment such as diarrhea and other gastrointestinal toxicities.

Neutropenia is also a frequent TEAE associated with abemaciclib and other CDK4 and 6 inhibitors [23]. In our pooled analysis, neutropenia was the most common Grade ≥ 3 AE; interestingly, incidence of neutropenia was not increased in patients >65 years, consistent with data reported from an FDA pooled analysis of CDK4 and 6 inhibitors in older women [5]. In fact, in our analysis, the ≥75 age group had the lowest incidence of neutropenia; this was also reflected by the lower rate of G-CSF/GM-CSF use in this age group.

VTEs are a known AE of special interest for abemaciclib. The incidence of VTE was higher in all age groups in the abemaciclib arm compared to the placebo arm in MONARCH 2 and 3. Of note, VTEs are not specific to abemaciclib but have been reported for CDK inhibitors as a class effect [24]. The higher incidence of VTE in the subgroup of patients ≥75, together with age being a risk factor, suggests that these patients should be more carefully monitored for early symptoms. A recent review by the FDA suggests that the occurrence of ILD/pneumonitis may also be a class effect [25]; rates of ILD/pneumonitis reported in MONARCH 2 and 3 are similar to those observed in studies of other CDK4 and 6 inhibitors. In our pooled analysis, ILD/pneumonitis was reported at similar frequency (~3.5%) across age groups in the abemaciclib + ET arm.

While abemaciclib (150 mg BID) + ET demonstrated a generally tolerable safety profile in older patients, with no new safety concerns compared to the overall MONARCH 2 and 3 populations [15, 16], higher rates of some AEs occurred in older patients. Patients in the 65–74 and ≥75 age groups were more likely to have had potentially confounding comorbidities including hypertension, gastrointestinal disorders, and metabolism/nutritional disorders. In addition, there were more patients with ECOG PS 1 in the two older subgroups; however, no other assessments were done to further characterize the fitness/frailty in the older patients, and we acknowledge that ECOG PS is not the best indicator of functional impairment in this population [26, 27]. Patients in the 2 older subgroups required more dose adjustments to manage AEs, which is in line with a previously published analysis of CDK4 and 6 inhibitors [5]. Of note, dose adjustment data from the pooled analysis were impacted by starting dose in MONARCH 2, especially the >75 age group that had the highest proportion of patients starting on the 200 mg dose (34.1%). The most frequent AE causing dose reductions was diarrhea, and the ≥75 subgroup had a modest increase (8%) over the <65 group. This could possibly be due to a more conservative management of the toxicities in older patients because of their additional burden of comorbidities and the clinical impact that even low-grade toxicities may have in these populations [28].

Abemaciclib + ET provided a consistent PFS benefit across all age groups with a clinically relevant magnitude of effect observed in all age groups as indicated by the lack of significance in the interaction p values. Overall, median PFS in patients receiving ET-only was shorter in older patients. For instance, in MONARCH 2, median PFS in the placebo arm was much lower in the older age group relative to the younger age group: 10.78 months in patients <65 versus 5.75 months in patients ≥75 years. In MONARCH 3, PFS in the placebo arm was also lower in older versus younger age groups but to a lesser extent: 13.97 months in patients <65 versus 9.11 months in patients ≥75 years. However, in patients 65–74 years old, median PFS in the placebo arm was much longer (24.20 months). Our analysis shows that the addition of abemaciclib to the ET backbone provides a PFS benefit across all age groups, including in the two older age groups. More research is warranted to better characterize the impact of age on safety and efficacy outcomes, using end points more relevant for older patients such as quality of life and maintenance of functional status [21]. Notably, a prospective phase II study (NCT04305834) is ongoing and designed to specifically estimate the incidence of grade 3 or higher toxicities attributed to abemaciclib monotherapy in adults aged 70 or older with HR+ metastatic breast cancer [29].

Taken together, our data suggest that while abemaciclib was generally well tolerated overall, clinicians should be aware of potentially higher incidence of gastrointestinal toxicities in older patients, including Grade 2/3 diarrhea. A proactive approach to treating older patients should include more careful monitoring of toxicities, including low grade adverse events, dose adjustments and use of supportive medication for gastrointestinal toxicities with the goal of maximizing abemaciclib tolerability. While age appears to be an important factor associated with higher rates of toxicity, age alone should not be considered in isolation when making treatment decisions. Future trials are warranted to further characterize the safety and efficacy of abemaciclib and other CDK4 and 6 inhibitors in broader older patient populations that are more representative of real-world settings.
